# Evidence Quality Assessment of Tai Chi Exercise Intervention in Cognitive Impairment: An Overview of Systematic Review and Meta-Analysis

**DOI:** 10.1155/2022/5872847

**Published:** 2022-04-25

**Authors:** Hongshuo Shi, Chengda Dong, Hui Chang, Lujie Cui, Mingyue Xia, Wenwen Li, Di Wu, Baoqi Yu, Guomin Si, Tiantian Yang

**Affiliations:** ^1^Shandong University of Traditional Chinese Medicine, Jinan, China; ^2^Shandong Provincial Hospital Affiliated to Shandong First Medical University, Jinan, China

## Abstract

**Background:**

Tai Chi (TC) exercise has recently received wide attention for its efficacy in the management of cognitive impairment. The purpose of this overview is to summarize the available evidence on TC treatment of cognitive impairment and assess its quality.

**Methods:**

We retrieved relevant systematic reviews/meta-analyses (SRs/MAs) from 7 databases from the time they were established to January 2, 2022. Two reviewers independently evaluated the methodological quality, risk of bias, report quality, and evidence quality of the included SRs/MAs on randomized controlled trials (RCTs). The tools used are Assessment System for Evaluating Methodological Quality 2 (AMSTAR-2), the Risk of Bias In Systematic (ROBIS) scale, the list of Preferred Reporting Items for Systematic Reviews And Meta-Analysis (PRISMA), and the Grading of Recommendations Assessment, Development, and Evaluation (GRADE) system.

**Results:**

This overview finally included 8 SRs/MAs. According to the results of AMSTAR-2, all included SRs/MAs were rated as very low quality. Based on the ROBIS tool, none of the SR/MA had a low risk of bias. In light of PRISMA, all SRs/MAs had reporting deficiencies. According to the GRADE system, there was only 1 high-quality piece of evidence.

**Conclusion:**

TC is a promising complementary and alternative therapy for cognitive impairment with high safety profile. However, in view of the low quality of the included SRs/MAs supporting this conclusion, high-quality evidence with a more rigorous study design and a larger sample size is needed before making a recommendation for guidance.

## 1. Introduction

As the aging population continues to grow, global public health is facing the serious problem of age-related cognitive decline. It is noteworthy that more and more people suffer from mild cognitive impairment (MCI) and dementia [1]. MCI occurs on a continuum from normal cognition to dementia, and individuals with MCI have a higher risk of dementia [2]. A recent report showed a 10%–25% incidence of MCI in people over 65 years of age [3], and the risk of dementia in MCI patients (10–15%) is much higher than in healthy older adults (1-2%) [4]. As cognitive performance declines, most individuals develop neuropsychiatric or behavioral [5] abnormalities in activities of daily living [6], ultimately resulting in a decline in quality of life (QoL) and an increased burden for family caregivers [7], and health professionals [8]. However, there is currently no drug treatment approved by the U.S. Food and Drug Administration to treat MCI or slow the long-term progression of MCI to dementia [9]. Therefore, complementary and alternative therapies have become a research hotspot in improving cognitive impairment in recent years [10].

In recent decades, increasing evidence suggests that exercise could be considered as a promising nonpharmacological intervention to improve cognitive performance [11]. As a traditional Chinese martial art, Tai Chi (TC) is a body-mind coordination exercise, and it perfectly integrates traditional philosophy and traditional Chinese medicine theory and pursues the unity of strength, shape, qi, and consciousness [12]. TC exercise mainly includes the stretching and relaxation of skeletal muscles, as well as various movements such as body coordination, regular breathing, and meditation [13]. TC has widely been accepted as a supplementary form of physical exercise in Western countries such as the United States and Britain [14], and there is now growing evidence that TC may help improve cognitive function and mental health in older adults with mild dementia [15, 16]. TC may be a potential treatment modality for patients with cognitive impairment.

Systematic reviews (SRs)/meta-analyses (MAs) are significant tools to conduct evidence-based clinical work. A growing number of SRs/MAs based on TC intervention for cognitive impairment suggest that TC can improve patients' cognitive function, delay the development of cognitive impairment, and improve the quality of life. However, without objective and comprehensive assessment of their methodological and evidentiary quality, it remains controversial whether these findings provide credible evidence for clinicians [17, 18]. This overview aimed to objectively and comprehensively evaluate the scientificity of TC exercise in the treatment of cognitively impaired SRs/MAs.

## 2. Methods

### 2.1. Research Methods and Protocol Registration

The overview of SRs/MAs was based on the guidelines specified in Cochrane Handbook [19], and other overviews with high-quality research methodology [20–22]. This overview protocol has been registered with the INPLASY website (Registration number: INPLASY202240055).

### 2.2. Eligibility Criteria

#### 2.2.1. Literature Inclusion Criteria

- Type of research
- This overview includes SRs/MAs of randomized controlled trials (RCTs) of the effects of TC exercise on cognitive impairment.
- Type of participants
- Subjects were patients diagnosed with MCI or dementia by any international or national standard.
- Type of intervention
- The intervention for the control group was conventional treatment (CT) or daily life activities, and the intervention for the experimental group was TC exercise or TC combined with the treatments received by the control group. CT includes health education, routine care, attention control, or medication.
- Types of outcomes
- At least one measure of cognitive domains was reported, such as global cognitive function, memory, executive function, attention, verbal fluency, and visuospatial function. Also, other assessment results obtained from relevant scales were included as well.

#### 2.2.2. Exclusion Criteria

(1) Animal studies and (2) network MAs, research protocols, narrative reviews, overviews, dissertation, and conference abstracts.

### 2.3. Data Sources and Search Strategy

Two researchers searched seven electronic databases for inception date up to January 2, 2022, including PubMed, Cochrane Library, EMBASE, Wanfang Database, CNKI, China Biomedicine (CBM), and Chongqing VIP, respectively. A literature search was carried out using a combination of key terms and free words, such as “Tai Chi,” “Cognitive Impairment,” “Systematic Review,” and “Meta-Analysis,” and the search strategy was finely adjusted according to different databases. The search strategy of PubMed database is shown in Table 1.

### 2.4. Literature Screening and Data Extraction

The literature screening (WW-L and LJ-C) and information extraction (H-C and MY-X) were independently performed by two researchers. The retrieved documents were imported into Endnote X9 document management software, and then, the duplicates were removed. The literature that potentially met the inclusion and exclusion criteria was then obtained by reading the titles and abstracts of the literature. Finally, we finalized the included MAs by reading the full text. All SRs/MAs were read by two independent researchers, and the following data were extracted from the SRs/MAs: first author, publication year, country, number of RCTs included, interventions for experimental and control groups, included RCT quality assessment tools, and main conclusion. The disagreement between the two researchers was resolved through discussion.

### 2.5. Quality Assessment for Inclusion in MAs

Two researchers (BQ-Y and D-W) independently assessed the methodological and evidence quality of the included SRs/MAs.

#### 2.5.1. Estimate of Methodological Quality

The methodological quality of the included SRs/MAs was assessed by the Assessment System for Evaluating Methodological Quality 2 (AMSTAR-2) [23]. Seven (2, 4, 7, 9, 11, 13, and 15) of the 16 items in the tool were critical areas.

#### 2.5.2. Estimate of Risk of Bias

The Risk of Bias In Systematic Review (ROBIS) [24] scale was used in this overview to evaluate the risk of bias of the inclusion of SRs/MAs. The scale was divided into three stages to assess the overall risk of bias in the inclusion of SRs/MAs.

#### 2.5.3. Estimate of Reporting Quality

The quality of each SR/MA report was evaluated by the list of Preferred Reporting Items for Systematic Reviews and Meta-Analysis (PRISMA) [25], which consisted of 27 items focusing on reporting methods and results that were incorporated into SRs/MAs.

#### 2.5.4. Assessment of Quality of Evidence

The quality of evidence for each SR/MA outcome was evaluated by The Grading of Recommendations Assessment, Development, and Evaluation (GRADE) [26], and five aspects will lead to the degradation of evidence quality, including limitations, inconsistencies, indirectness, imprecision, and publication bias. Evidence with less than one degrading factor (including one) was rated as high-quality evidence, while evidence with two degrading factors was rated as moderate quality, three degrading factors as low quality, and more than three (including three) degrading factors as very low quality.

## 3. Results

### 3.1. Results on Literature Search and Selection

**Figure 1:**
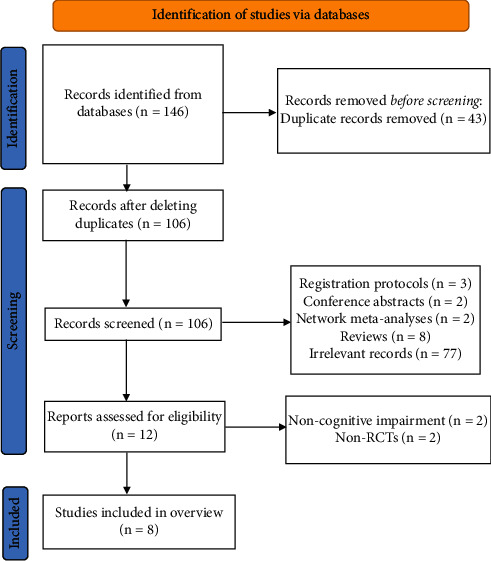
The flowchart of the screening process.

Through our search strategy, a total of 146 articles were identified. After removing 43 duplicate articles, the researchers screened the remaining 103 articles by reading titles and abstracts. Subsequently, the 12 articles were obtained. After reading the full text, it was found that two articles were not about SRs/MAs in RCTs, and two SRs/MAs were not about people with cognitive impairment. Finally, a total of 8 SRs/MAs [27–34] were finally included in this overview. The process of study selection is shown in Figure 1.

### 3.2. Description of the Included SRs/MAs

The characteristics included in the overview are shown in Table 2. These SRs/MAs were all published between 2017 and 2021, 5 [27–31] of which were in English, and the remaining 3 [32–34] were in Chinese, and all were written by Chinese authors. The number of RCTs was between 3 and 19, and the sample size was between 378 and 1,970. In 5 SRs/MAs [27–31], the intervention method for the control group was CT or daily life activities, while that for the experimental group was TC or TC combined with the intervention methods for the control group. In 3 SRs/MAs [32–34], the intervention method for the control group was CT or daily life activities, while that for the experimental group was TC exercise. In terms of quality evaluation scales, 6 SRs/MAs [27, 29, 30, 32–34] used the Cochrane risk of bias standard, and 2 SRs/MAs [28, 31] used the Physiotherapy Evidence Database scale.

### 3.3. Results of the Methodological Quality

By using AMSTAR-2 to assess the methodological quality, all SRs/MAs were considered to be of very low quality because more than one key item was missing from the included SRs/MAs. The restrictions came from the following items: Item 2 (only 2 SRs/MAs [29, 30] have registered protocol), Item 7 (the list of excluded studies was not mentioned by any SR/MA), Item 10 (none reported the funding of RCTs included in SRs/MAs), and Item 15 (only one SR/MA [31] conducted publication bias assessment or discussed their impact on SR/MA). The AMSTAR-2 assessment breakdown for each SR/MA is shown in Table 3.

### 3.4. Risk of Bias of the Included SRs/MAs

By means of ROBIS, we evaluated the relevance of Phase 1 of the research theme, and all SRs/MAs were rated as low risk of bias. In Phase 2, Domain 1, all SRs/MAs were rated as low risk of bias. In Domain 2, 5 SRs/MAs [27, 28, 30, 31, 34] were rated as low risk. In Domain 3, 6 SRs/MAs [27, 28, 31–34] of which were rated as low risk of bias, and none of one SR/MA was rated as low risk of bias in Domain 4. In Phase 3, all SRs/MAs were rated as low risk of bias. The included ROBIS evaluation details of SRs/MAs are shown in Table 4.

### 3.5. Report Quality of the Included SRs/MAs

Table 5 lists the details of the PRISMA checklist for each SR/MA. Although the title, abstract, introduction, and discussion were reported in full, some reporting flaws were still found in other sections. In the methods section, Item 7 (search strategy), Item 14 (reporting bias assessment), and Item 15 (certainty assessment) were insufficiently reported (<50%). In the results section, Item 16b (study selection), Item 20d (results of syntheses), Item 21 (reporting biases), and Item 22 (certainty of evidence) were reported as less than 50%. In addition to this, the Item 24 a, b, c (registration and protocol) reports for the included SRs/MAs were missing.

### 3.6. Evidence Quality of the Included SRs/MAs

The 42 outcomes included in the 8 SRs/MAs were assessed using the GRADE system. In the evaluation results based on the outcome indicators, 1 SR/MA was rated high, 8 moderate, 19 low, and 14 very low in terms of the quality of evidence. Publication bias (*n* = 36) was the most common downgrading factor, followed by imprecision (*n* = 24), inconsistency (*n* = 21), risk of bias (*n* = 12), and indirectness (*n* = 0) (Table 6).

### 3.7. Summary Results of the Included Studies

The result indicators extracted from the included studies are listed in Table 6.

#### 3.7.1. Global Cognitive Function

All the included SRs/MAs reported the effect of TC on the overall cognitive function of the included population, and the results of 7 SRs/MAs [27–33] indicated that TC could significantly improve the overall cognitive function of the cognitively impaired population.

#### 3.7.2. Memory and Learning

7 SRs/MAs [28–34] reported the effect of TC on memory and learning, and the results of 6 SRs/MAs [28–32, 34] indicated that TC could significantly improve the memory and learning performance in people with cognitive impairment.

#### 3.7.3. Visuospatial Ability

4 SRs/MAs [28, 30, 31, 34] reported the effect of TC on visuospatial ability of which 3 SRs/MAs [28, 30, 34] reported that TC could significantly improve the visuospatial ability of patients with cognitive impairment.

#### 3.7.4. Executive Function

5 SRs/MAs [27, 29–31, 34] reported the effect of TC on executive function of which 3 SRs/MAs [27, 30, 31] reported that TC could significantly improve the executive function of patients with cognitive impairment.

#### 3.7.5. Verbal Fluency

3 SRs/MAs [28, 30, 34] reported the effect of TC on verbal fluency of which 2 SRs/MAs [28, 34] reported that TC could significantly improve the verbal fluency of patients with cognitive impairment.

#### 3.7.6. Psychological Evaluation

3 SRs/MAs [30, 32, 34] reported on psychological assessments, and only 1 SR/MA [32] showed that TC could improve the mental activity of patients with cognitive impairment.

#### 3.7.7. Other Outcome Indicators

A SR/MA [28] reported that TC can significantly improve mental speed and attention and ideas, abstraction, figural creations, and mental flexibility in patients with cognitive impairment. A separate SR/MA [30] reported that TC could improve the physical activity and attention [31] of patients.

#### 3.7.8. Adverse Reactions

The narrative descriptions in 5 SRs/MAs [27–31] indicated that TC was a safe treatment modality.

## 4. Discussion

Currently, drug treatments have limited effectiveness in improving cognition or slowing disease progression [35]. Physical activity is a well-studied behavioral intervention for cognitive function [36], and TC may be a good one. A literature search revealed that although several SRs/MAs on the impact of TC on cognitive impairment have been published, the quality of these publications has not been assessed. Therefore, we carried out this overview to evaluate the multiple SRs/MAs that meet the inclusion criteria in a bid to provide clinicians with higher-quality evidence.

### 4.1. Summary of the Main Findings

This is the first overview of SRs/MAs on the effects of TC on cognitive impairment, including 8 SRs/MAs on TC for cognitive impairment, published between 2011 and 2021, and 7 of the SRs/MAs (7/8, 87.5%) were published after 2020. This may indicate that TC, as a complementary and alternative therapy for cognitive impairment, has drawn increasing attention from people.

As indicated by the assessment for method quality, report quality, risk of bias, and evidence quality, the included 8 SRs/MAs were not satisfactory. In AMSTAR-2, all the included SRs/MAs are considered to be of very low quality, and the main defects are pointed out as follows: (1) only two SRs/MAs [29, 30] were registered with the study protocol, which may affect its standardization and sophistication, and increase the possibility of selective reporting bias; (2) none of the SR/MA provided a list of excluded literature, which may reduce the transparency of the SRs/MAs and affect the credibility of the results; (3) only one SR/MA [31] assessed publication bias in the included RCTs, which would reduce confidence in the results. In addition, this was also related to the insufficient number of RCTs included in the relevant outcome measures; (4) in addition, no SR/MA reporting was included in the RCT funding resources, which may increase bias in clinical trials since the results of corporate-funded studies may be biased in favor of the funder. All of the above methodological flaws limit the accuracy of SRs/MAs. In the ROBIS assessment, insufficient assessment of publication bias was the main reason for the high risk of final results, which was consistent with the AMSTAR-2 scale. Moreover, the absence of sensitivity analysis was also an important factor leading to high risk of bias, which would affect the stability of the SRs/MAs results. Regarding the results of the PRISMA checklist, lack of protocol registration and publication bias in SRs/MAs was the main cause of underreporting, as shown in AMSTAR-2. However, none of the SRs/MAs provided comprehensive search strategies, which reduced the reproducibility and credibility of the study.

For GRADE, publication bias was the most common downgrading factor included in SRs/MAs. Insufficient assessment of publication bias in the outcome measures was the main downgrading factor, which was also related to the inadequate number of RCTs included in the relevant outcome measures. In addition, the insufficient study population included in a single effect size was also an important reason for the decline in the quality of the evidence. Although almost all SRs/MAs showed that TC had a positive effect on cognitive function in patients with cognitive impairment, the conclusions of SRs/MAs may deviate from the real results due to the inadequate methodological and evidence quality of the included studies. Caution should be exercised in recommending TC as a complementary intervention for cognitive impairment.

### 4.2. Implications for Future Study

Our overview may have some reference value for future research. Authors should pay attention to the registration of research protocols before proceeding with SRs/MAs to ensure the rigor of their procedures. In terms of literature search and selection, information on excluded literature and complete search strategy for all databases should be listed and elaborated on to ensure transparency. In the quantitative calculation of effect size, care should be taken to exclude the results of a single study one by one to ensure the stability of the results. In addition, a complete assessment of publication bias would also improve the accuracy of the meta-analysis results. TC is not only easy to learn and practice but also has many advantages in physiology and psychology, and it has clinical significance for further research. Although TC originated from traditional Chinese medicine theory, the duration, frequency, and mode of TC movement vary greatly in different studies. Therefore, we propose to use a standardized TC training program, including fixed duration, frequency, and pattern, to better study the impact of TC on cognitive performance. In addition, the assessment of cognitive function should identify areas of cognition specifically improved by TC in patients with cognitive impairment, as indicated by as physiological outcomes, such as circulating biochemical markers and neuroimaging structure and function. With the evolution of evidence-based medicine, it is hoped that researchers will continue to promote the standardization of relevant individual RCTs in the future. A well-designed, rigorously implemented, and complete reporting RCT with complete reporting can minimize or avoid bias. It is the gold standard for evaluating interventions [37].

### 4.3. Strength and Limitations

Our overview is the first to use AMSTAR-2, ROBIS, PRISMA, and GRADE to evaluate SRs/MAs regarding the impact of TC on cognitive impairment. Based on the current results, TC may be an effective adjunctive replacement therapy for cognitive impairment. Furthermore, the evaluation process revealed clear limitations of the current relevant SRs/MAs and RCTs, which may help guide high-quality clinical studies in the future. However, this overview has certain limitations because of the subjectivity of the assessment. Although our assessments were reviewed by two independent assessors, different assessors may have their own judgment on each factor, so the results may vary.

## 5. Conclusion

Based on current evidence, TC appears to have a positive effect on cognitive impairment with a high safety profile. However, the low quality of the SRs/MAs supporting these results is concerning, and we should therefore approach this conclusion with caution. In the future, RCTs with more stringent TC interventions for cognitive impairment should be performed. At the same time, more rigorous, standardized, and comprehensive SRs/MAs in related fields are needed to provide stronger evidence.

## Figures and Tables

**Table 1:** Search strategy for the PubMed database.

Query	Search term
#1	“Tai Ji” [Mesh]
#2	“Tai-ji” OR “Tai Chi” OR “Chi, Tai” OR “Tai Ji Quan” OR “Ji Quan, Tai” OR “Quan, Tai Ji” OR “Taiji” OR “Taijiquan” OR “T'ai Chi” OR “Tai Chi Chuan” OR “Taiji”
#3	#1 OR #2
#4	“Cognitive Dysfunction” [Mesh]
#5	“Cognitive Dysfunctions” OR “Dysfunction, Cognitive” OR “Dysfunctions, Cognitive” OR “Cognitive Impairments” OR “Cognitive Impairment” OR “Impairment, Cognitive” OR “Impairments, Cognitive” OR “Mild Cognitive Impairment” OR “Cognitive Impairment, Mild” OR “Cognitive Impairments, Mild” OR “Impairment, Mild Cognitive” OR “Impairments, Mild Cognitive” OR “Mild Cognitive Impairment” OR “Mild Neurocognitive Disorder” OR “Disorder, Mild Neurocognitive” OR “Disorders, Mild Neurocognitive” OR “Mild Neurocognitive Disorders” OR “Neurocognitive Disorder, Mild” OR “Neurocognitive Disorders, Mild” OR “Cognitive Decline” OR “Cognitive Dysfunction” OR “Cognitive Declines” OR “Decline, Cognitive” OR “Declines, Cognitive” OR “Mental Deterioration” OR “Deterioration, Mental” OR “Deteriorations, Mental” OR “Mental Deteriorations”
#6	#4 OR #5
#7	Meta-Analysis as Topic [Mesh]
#8	“Systematic review” OR “meta-analysis” OR “meta analysis” OR “meta-analyses” OR “Review, Systematic”
#9	#7 OR #8
#10	#3 AND #6 AND #9

**Table 2:** Characteristics of the included SRs/MAs.

Author, year (country)	Trials (subjects)	Intervention group	Control group	Quality assessment	Main results
Liu et al., 2021 (China) [27]	10 (580)	TC, TC + CT	CT and daily life activities	Cochrane criteria	TC may have a positive effect on cognitive function improvement in middle-aged and elderly patients with cognitive impairment
Yang et al., 2020 (China) [28]	11 (1,061)	TC, TC + CT	CT and daily life activities	Physiotherapy Evidence Database scale	TC may be beneficial in improving cognitive function in older adults with MCI. However, good RCTs need to be rigorously designed and reported
Gu et al., 2021 (China) [29]	9 (827)	TC, TC + CT	CT and daily life activities	Cochrane criteria	Evidence that supports the efficacy of TC in older adults with cognitive impairment is limited. Tai Chi appears to be a safe exercise that leads to better changes in cognitive function scores
Lin et al., 2021 (China) [30]	7 (1,265)	TC, TC + CT	CT and daily life activities	Cochrane criteria	This meta-analysis demonstrates that TC has a positive clinical effect on cognitive function (overall cognitive function, memory and learning, and executive function) and physical abilities in older adults with MCI, and provides a feasible approach for MCI management
Cai et al., 2020 (China) [31]	19 (1,970)	TC and TC + CT	CT and daily life activities	Physiotherapy Evidence Database scale	TC is a promising approach to improve overall cognitive function, memory, executive function, attention, and language fluency in older adults with cognitive impairment
Li et al., 2021 (China) [32]	11 (1,234)	TC	CT and daily life activities	Cochrane criteria	TC has a certain positive effect on the cognitive function of MCI patients, but the research on the rehabilitation effect should still be increased
Zhang et al., 2017 (China) [33]	3 (378)	TC	CT and daily life activities	Cochrane criteria	TC exercise has a good effect on improving the cognitive function of the elderly with cognitive impairment
Zhang et al., 2020 (China) [34]	7 (1,068)	TC	CT and daily life activities	Cochrane criteria	TC can improve memory and visuospatial function in the elderly with mild cognitive impairment, but there is no significant improvement in indicators such as overall cognitive function, executive ability, language fluency, and depression

**Table 3:** Result of the AMSTAR-2 assessments.

Author, year (country)	Q1	Q2	Q3	Q4	Q5	Q6	Q7	Q8	Q9	Q10	Q11	Q12	Q13	Q14	Q15	Q16	Overall quality
Liu et al., 2021 (China) [27]	Y	PY	Y	Y	Y	Y	N	Y	Y	N	Y	Y	Y	Y	N	Y	VL
Yang et al., 2020 (China) [28]	Y	PY	Y	Y	Y	Y	N	Y	Y	N	Y	Y	Y	Y	N	Y	VL
Gu et al., 2021 (China) [29]	Y	Y	Y	PY	N	Y	N	Y	Y	N	Y	Y	Y	Y	N	Y	VL
Lin et al., 2021 (China) [30]	Y	Y	Y	Y	N	N	N	Y	Y	N	Y	Y	Y	Y	N	Y	VL
Cai et al., 2020 (China) [31]	Y	PY	Y	Y	Y	Y	N	Y	Y	N	Y	Y	Y	Y	Y	Y	VL
Li et al., 2021 (China) [32]	Y	PY	Y	PY	Y	Y	N	Y	Y	N	Y	Y	Y	Y	N	Y	VL
Zhang et al., 2017 (China) [33]	Y	PY	Y	PY	Y	Y	N	Y	Y	N	Y	Y	N	Y	N	Y	VL
Zhang et al., 2020 (China) [34]	Y	PY	Y	Y	Y	Y	N	Y	Y	N	Y	Y	Y	Y	N	N	VL

*Note.* Y, yes; PY, partial yes; N, no; VL, very low; L, low; key items are marked in red; Item 1, whether the research question and inclusion criteria include PICO elements; Item 2, whether to report systematic review research methods that were determined prior to implementation, and whether to report inconsistencies with the proposal; Item 3, did the authors explain why the systematic review was chosen for inclusion in the type of study design; Item 4, whether the authors used a comprehensive literature search strategy; Item 5, whether the literature screening was completed by 2 people independently; Item 6, whether the data extraction was completed independently by 2 people; Item 7, whether a list of excluded literature and reasons for exclusion is provided; Item 8, whether the authors describe the essential characteristics of the included studies in sufficient detail; Item 9, whether the authors used reasonable tools to assess the risk of bias of the included studies; Item 10, whether the authors reported funding for the studies included in this systematic review; Item 11, if a meta-analysis was performed, whether the authors used appropriate statistical methods to pool the results; Item 12, if meta-analyses were performed, whether the authors considered the potential impact of the included studies' risk of bias on meta-analyses or other evidence integration; Item 13, whether the authors considered the risk of bias of the included studies when interpreting/discussing the results of the systematic review; Item 14, whether the authors gave a satisfactory explanation or discussion of the heterogeneity in the results of the systematic review; Item 15, if quantitative synthesis was performed, whether the authors adequately investigated publication bias and discussed its possible impact on the findings; Item 16, whether the authors reported any potential conflicts of interest, including any funding received to conduct the systematic review.

**Table 4:** Results of the ROBIS assessments.

Author, year (country)	Phase 1	Phase 2				Phase 3
	Assessing relevance	Domain 1: study eligibility criteria	Domain 2: identification and selection of studies	Domain 3: collection and study appraisal	Domain 4: synthesis and findings	Risk of bias in the review
Liu et al., 2021 (China) [27]	√	√	√	√	×	√
Yang et al., 2020 (China) [28]	√	√	√	√	×	√
Gu et al., 2021 (China) [29]	√	√	×	×	×	√
Lin et al., 2021 (China) [30]	√	√	√	×	×	√
Cai et al., 2020 (China) [31]	√	√	√	√	×	√
Li et al., 2021 (China) [32]	√	√	×	√	×	√
Zhang et al., 2017 (China) [33]	√	√	×	√	×	×
Zhang et al., 2020 (China) [34]	√	√	√	√	×	√

Note: √, low risk; ×, high risk.

**Table 5:** Results of the PRISMA checklist.

Section/topic		Items	Liu et al., 2021 (China) [27]	Yang et al., 2020 (China) [28]	Gu et al., 2021 (China) [29]	Lin et al., 2021 (China) [30]	Cai et al., 2020 (China) [31]	Li et al., 2021 (China) [32]	Zhang et al., 2017 (China) [33]	Zhang et al., 2020 (China) [34]	Number of yes or partially yes (%)
Title	Title	Item 1	Y	Y	Y	Y	Y	Y	Y	Y	100
Abstract	Abstract	Item 2	PY	PY	PY	PY	PY	PY	PY	PY	100
Introduction	Rationale	Item 3	Y	Y	Y	Y	Y	Y	Y	Y	100
	Objectives	Item 4	Y	Y	Y	Y	Y	Y	Y	Y	100
Methods	Eligibility criteria	Item 5	Y	Y	Y	Y	Y	Y	Y	Y	100
	Information sources	Item 6	Y	Y	Y	Y	Y	Y	Y	Y	100
	Search strategy	Item 7	PY	N	PY	N	N	N	N	PY	37.50
	Selection process	Item 8	Y	Y	N	N	Y	Y	Y	Y	75
	Data collection process	Item 9	Y	Y	Y	N	Y	Y	Y	Y	87.50
	Data items	Item 10 (a)	Y	Y	Y	Y	Y	Y	Y	Y	100
		Item 10 (b)	PY	PY	PY	PY	PY	PY	PY	PY	100
	Study risk of bias assessment	Item 11	Y	Y	Y	Y	Y	Y	Y	Y	100
	Effect measures	Item 12	Y	Y	Y	Y	Y	Y	Y	Y	100
	Synthesis methods	Item 13 (a)	Y	Y	Y	Y	Y	Y	Y	Y	100
		Item 13 (a)	Y	Y	Y	Y	Y	Y	Y	Y	100
		Item 13 (c)	Y	Y	Y	Y	Y	Y	Y	Y	100
		Item 13 (d)	Y	Y	Y	Y	Y	Y	Y	Y	100
		Item 13 (e)	Y	N	Y	Y	N	N	Y	Y	62.50
		Item 13 (f)	Y	N	N	Y	N	N	Y	Y	50
	Reporting bias assessment	Item 14	N	N	N	N	Y	N	N	N	12.50
	Certainty assessment	Item 15	N	N	N	N	N	N	N	N	0
Results	Study selection	Item 16 (a)	Y	Y	Y	Y	Y	Y	Y	Y	100
		Item 16 (b)	N	Y	N	Y	N	N	N	N	25
	Study characteristics	Item 17	Y	Y	Y	Y	Y	Y	Y	Y	100
	Risk of bias in studies	Item 18	Y	Y	Y	Y	Y	Y	Y	Y	100
	Results of individual studies	Item 19 (a)	Y	Y	Y	Y	Y	Y	Y	Y	100
		Item 19 (b)	Y	Y	Y	Y	Y	Y	Y	Y	100
	Results of syntheses	Item 20 (a)	Y	Y	Y	Y	Y	Y	Y	Y	100
		Item 20 (b)	Y	Y	Y	Y	Y	Y	Y	Y	100
		Item 20 (c)	Y	Y	Y	Y	Y	Y	Y	N	87.50
		Item 20 (d)	Y	N	N	N	N	N	N	Y	25
	Reporting biases	Item 21	N	N	N	N	Y	N	N	N	12.50
	Certainty of evidence	Item 22	N	N	N	N	N	N	N	N	0
Discussion	Discussion	Item 23 (a)	Y	Y	Y	Y	Y	Y	Y	Y	100
		Item 23 (b)	Y	Y	Y	Y	Y	Y	Y	Y	100
		Item 23 (c)	Y	Y	Y	Y	Y	Y	Y	Y	100
		Item 23 (d)	Y	Y	Y	Y	Y	Y	Y	Y	100
Other information	Registration and protocol	Item 24(a)	N	N	Y	Y	N	N	N	N	25
		Item 24 (b)	N	N	Y	Y	N	N	N	N	25
		Item 24 (c)	N	N	N	N	N	N	N	N	0
	Support	Item 25	Y	Y	Y	Y	Y	Y	Y	N	87.50
	Competing interests	Item 26	Y	Y	Y	Y	Y	Y	Y	Y	100
	Availability of data, code, and other materials	Item 27	Y	Y	Y	Y	Y	Y	Y	Y	100

*Note.* Y, yes; N, no; PY, partially yes.

**Table 6:** Results of evidence quality.

Author, year (country)	Outcomes	Studies (participants)	Limitations	Inconsistency	Indirectness	Imprecision	Publication bias	Relative effect (95% CI)	Heterogeneity (%)	Quality
Liu et al., 2021 (China) [27]	MoCA (global cognitive function)	5 (344)	0 (1)	−1 (2)	0	−1 (3)	−1 (4)	WMD = 3.23, 95% CI (1.88, 4.58)^*∗*^	I2 = 92	Very low
	MMSE (global cognitive function)	3 (187)	0	−1 (2)	0	−1 (3)	−1 (4)	WMD = 3.69, 95% CI (0.31, 7.08)^*∗*^	I2 = 83	Very low
	TMT-B (executive function)	2 (147)	0	0	0	−1 (3)	−1 (4)	WMD = −13.69, 95% CI (−21.64, −5.74)^*∗*^	I2 = 0	Low
Yang et al., 2020 (China) [28]	Global cognitive function	5 (858)	0	−1 (2)	0	0	−1 (4)	SMD = 0.40, 95% CI (0.24, 0.55)^*∗*^	I2 = 63	Low
	Memory and learning	7 (855)	0	−1 (2)	0	0	−1 (4)	SMD = 0.37, 95% CI (0.24, 0.51)^*∗*^	I2 = 67	Low
	Mental speed and attention	6 (929)	0	−1 (2)	0	0	−1 (4)	SMD = 0.51, 95% CI (0.31, 0.71)^*∗*^	I2 = 84	Low
	Ideas, abstraction, figural creations, and mental flexibility	6 (782)	0	0	0	0	−1 (4)	SMD = 0.29, 95% CI (0.16, 0.42)^*∗*^	I2 = 0	Moderate
	Visuospatial ability	3 (192)	0	0	0	−1 (3)	−1 (4)	SMD = 0.29, 95% CI (0.10, 0.48)^*∗*^	I2 = 0	Low
Gu et al., 2021 (China) [29]	MMSE (global cognitive function)	6 (673)	−1 (1)	−1 (2)	0	0	−1 (4)	WMD = 1.52, 95% CI (0.90, 2.14)^*∗*^	I2 = 63	Very low
	MoCA (global cognitive function)	3 (244)	−1 (1)	−1 (2)	0	−1 (3)	−1 (4)	WMD = 3.5, 95% CI (0.76, 6.24)^*∗*^	I2 = 92	Very low
	CDR (global cognitive function)	2 (285)	−1 (1)	0	0	−1 (3)	−1 (4)	WMD = −0.55, 95% CI (−0.80, −0.29)^*∗*^	I2 = 0	Very low
	LMD (memory and learning)	3 (435)	−1 (1)	−1 (2)	0	0	−1 (4)	WMD = 1.10, 95% CI (0.04, 2.16)^*∗*^	I2 = 77	Very low
	DSF (executive function)	2 (287)	−1 (1)	−1 (2)	0	−1 (3)	−1 (4)	WMD = 0.53, 95% CI (−0.65, 1.71)	I2 = 64	Very low
	DSB (executive function)	2 (287)	−1 (1)	0	0	−1 (3)	−1 (4)	WMD = −0.1, 95% CI (−0.38, 0.19)	I2 = 0	Very low
Lin et al., 2021 (China) [30]	Global cognitive function	2 (272)	0	0	0	0	−1 (4)	WMD = -2.24, 95% CI (−3.51, −0.97)^*∗*^	I2 = 0	Moderate
	Memory and learning	3 (126)	0	−1 (2)	0	−1 (3)	−1 (4)	SMD = 0.83, 95% CI (0.22, 1.45)^*∗*^	I2 = 57	Very low
	Visuospatial ability	2 (85)	0	0	0	−1 (3)	−1 (4)	WMD = 3.15, 95% CI (0.74, 5.56)^*∗*^	I2 = 0	Low
	Executive function	3 (376)	0	0	0	−1 (3)	−1 (4)	WMD = 0.32, 95% CI (0.03, 0.61)^*∗*^	I2 = 0	Low
	Physical activity	2 (53)	0	0	0	−1 (3)	−1 (4)	WMD = 18.78, 95% CI (10.80, 26.76)^*∗*^	I2 = 0	Low
	Psychological assessment	2 (272)	0	0	0	−1 (3)	−1 (4)	WMD = 0.17, 95% CI (−0.62, 0.96)	I2 = 0	Low
Cai et al., 2020 (China) [31]	Global cognitive function	12 (1,738)	0	−1 (2)	0	0	0	SMD = 0.41, 95% CI (0.33, 0.48)^*∗*^	I2 = 67	Moderate
	Memory function	16 (1,708)	0	−1 (2)	0	0	0	SMD = 0.31, 95% CI (0.22, 0.39)^*∗*^	I2 = 69	Moderate
	Executive function	9 (1,586)	0	−1 (2)	0	0	0	SMD = 0.33, 95% CI (0.25, 0.42)^*∗*^	I2 = 77	Moderate
	Verbal fluency	5 (1,325)	0	0	0	0	0	SMD = 0.27, 95% CI (0.13, 0.41)^*∗*^	I2 = 0	High
	Attention	6 (1,479)	0	−1 (2)	0	0	0	SMD = 0.25, 95% CI (0.17, 0.34)^*∗*^	I2 = 96	Moderate
	Visual space function	3 (192)	0	−1 (2)	0	−1 (3)	0	SMD = 0.03, 95% CI (−0.28, 0.33)	I2 = 55	Low
Li et al., 2021 (China) [32]	GDS (psychological assessment)	2 (110)	−1 (1)	0	0	−1 (3)	−1 (4)	WMD ＝ −2.81, 95% CI (−3.48, −2.14)^*∗*^	I2 = 45	Very low
	DSF (executive function)	2 (355)	0	−1 (2)	0	−1 (3)	−1 (4)	WMD = 1.22, 95% CI (−0.68, 3.12)	I2 = 82	Very low
	DSB (executive function)	3 (620)	0	0	0	−1 (3)	−1 (4)	WMD = 0.17, 95% CI (−0.03, 0.36)	I2 = 18	Low
	MoCA (global cognitive function)	2 (136)	−1 (1)	−1 (2)	0	−1 (3)	−1 (4)	WMD = -1.58, 95% CI (−9.79, 6.64)	I2 = 97	Very low
	AVLT (memory and learning)	2 (123)	−1 (1)	−1 (2)	0	−1 (3)	−1 (4)	WMD ＝ 1.27，95% CI (0.31, 2.23)^*∗*^	I2 = 51	Very low
	LMD (memory and learning)	3 (660)	0	−1 (4)	0	0	−1 (4)	WMD ＝ 2.26, 95% CI (0.35, 4.16)^*∗*^	I2 = 93	Low
	MMSE (global cognitive function)	4 (704)	0	0	0	0	−1 (4)	WMD = 0.93, 95% CI (0.40, 1.47)^*∗*^	I2 = 0	Moderate
Zhang et al., 2017 (China) [33]	Global cognitive function	3 (678)	−1 (1)	0	0	0	−1 (4)	WMD = 0.91, 95% CI (0.37, 1.46)^*∗*^	I2 = 0	Low
	Verbal fluency	2 (654)	−1 (1)	0	0	0	−1 (4)	WMD = 2.17, 95% CI (0.88, 3.45)^*∗*^	I2 = 0	Low
	Memory function	2 (654)	−1 (1)	−1 (2)	0	−1 (3)	−1 (4)	WMD = 0.16, 95% CI (−0.14, 0.45)	I2 = 55	Very low
Zhang et al., 2020 (China) [34]	Global cognitive function	5 (785)	0	0	0	−1 (3)	−1 (4)	WMD = 0.29, 95% CI (−0.16, 0.74)	I2 = 0	Low
	Memory function	4 (726)	0	0	0	0	−1 (4)	WMD = 0.37, 95% CI (0.13, 0.61)^*∗*^	I2 = 7	Moderate
	Executive function	4 (726)	0	0	0	−1 (3)	−1 (4)	WMD = 0.03, 95% CI (−0.16, 0.22)	I2 = 0	Low
	Verbal fluency	2 (594)	0	0	0	−1 (3)	−1 (4)	WMD = 0.47, 95% CI (−0.76, 1.70)	I2 = 0	Low
	Visual space function	4 (726)	0	−1 (2)	0	0	−1 (4)	SMD = 0.57, 95% CI (0.23, 0.91)^*∗*^	I2 = 75	Low
	Psychological assessment	4 (730)	0	0	0	−1 (3)	−1 (4)	SMD = 0.00, 95% CI (−0.14, 0.15)	I2 = 0	Low

*Note.* (1) The included studies had a large bias in methodology such as randomization, allocation concealment, and blinding. (2) The confidence interval overlapped less or the I2 value of the combined results was larger. (3) The sample size from the included studies did not meet the optimal sample size or the 95% confidence interval crossed the invalid line. (4) The funnel chart was asymmetry. ^*∗*^The 95% confidence interval did not cross the invalid line. MoCA, Montreal Cognitive Assessment Scale; MMSE, mini-mental state examination; TMT-B, trail marking test B; CDR, clinical dementia rating; DSF, digit span forward; DSB, digit span backward; LMD, Logical Memory Delayed Recall Score; GDS, Geriatric Depression Scale; AVLT, auditory verbal learning test.

## Data Availability

The datasets analyzed during the current study are available from the corresponding author on reasonable request.
